# Myomucosal Lip Island Flap for Reconstruction of Small to Medium Lower Lip Defects

**DOI:** 10.1089/fpsam.2020.0068

**Published:** 2020-05-15

**Authors:** Simon Madorsky, Orr Meltzer

**Affiliations:** Skin Cancer and Reconstructive Surgery Center (SCARS Center), Newport Beach, California, USA.

## Abstract

**Importance:** Reconstruction of small to medium lower lip defects commonly includes mucosal advancement and wedge excision with primary closure, resulting in aesthetic complications such as lip flattening, shortening, and loss of the vermilion roll. The myomucosal lip island flap offers an alternative that preserves lower lip appearance and function.

**Objective:** To describe the lateral myomucosal lip island flap and its indications for the reconstruction of small to medium lower lip defects.

**Design, Setting, and Participants:** A retrospective chart analysis of patients from 2014 to 2019 was done. Participants include all consecutive patients of the senior author who had the myomucosal lip island flap employed in the lower lip from 2014 when the senior author began employing this technique, including 20 patients. Patient demographics, surgical indications, surgical defect bridging distances, flap advancing distances, functional complications, and aesthetic outcomes were reported. IRB approval was obtained from St. Joseph Health Center for Clinical Research and surgeries were performed at a private tertiary referral center—Skin Cancer and Reconstructive Surgery Center—by the senior author in a multispecialty practice.

**Main Outcomes and Measures:** Location and flaps utilized to reconstruct the defect were reported. Lateral advancing distance and overall bridging distance were measured. Functional complications, if any, were reported. Appearance rating after the first stage was assessed.

**Results:** This case series included 20 patients with lower lip defects reconstructed with myomucosal lip island flaps. The average bridging distance (width of defect) was 1.7 cm (minimum 1.0 cm, maximum 2.8 cm). Of 18 patients with available postoperative photographs, 4 cases (22%) had mild vermilion inferior retraction, 1 case (6%) had mild contour irregularity, and 1 case (6%) had visible white scar in the red lip.

**Conclusions and Relevance:** The myomucosal lip island flap is a reliable technique for reconstruction of small to medium lower lip defects, preserving lip fullness and the vermilion roll.

**Key Points**

**Question:** What are the indications, limitations, and aesthetic outcomes of the myomucosal lip island flap for lower lip reconstruction?

**Findings:** Myomucosal lip island flap can effectively treat defects from 1 to 2.8 cm extending deeply into the lip with excellent cosmetic results.

**Meaning:** The myomucosal lip island flap is an effective alternative to mucosal advancement and wedge excision with primary closure for small to medium lower lip defects.

## Introduction

Reconstruction of superficial lower lip defects inside the vermilion line has traditionally been performed with mucosal advancement. Mucosal excision and closure were introduced into the literature in Germany in the mid 1800s.^1–3^ In the 20th century, several authors have more specifically described mucosal advancement in the English language literature.^4–6^ This technique involves sacrificing surrounding red lip mucosa as the flap is advanced posterior to anterior. The posterior tension of advancement effaces the lip projection at the vermilion border, flattening the aesthetic fullness of the lower lip.^7^

This mucosal advancement was modified by Kolhe and Leonard in 1988,^8^ to include muscle and inferior labial artery within the leading flap edge. This myomucosal flap advancement lessened vermilion effacement while still sacrificing the surrounding mucosa.

Deeper tumors or defects of the lip extending into the orbicularis oris muscle require more than a mucosal flap closure. Defects less than one-third the width of the lip are treated with primary closure and a V or W wedge excision. These techniques sacrifice muscle and skin below the defect and cause horizontal shortening of the lip.^9,10^ Deep muscle defects have also been addressed with vertical V-Y myocutaneous flap advancement. This tissue-sparing technique is combined with various mucosal flaps to reconstruct the vermilion portion of the lip.^11,12^

Mohs surgery has changed the nature of most lower lip skin cancer defects by limiting the defect size. The older lower lip wedge techniques resect a much larger portion of the lower lip than is necessary. Thus, wedge excisions for carcinoma have become less common with the availability of Mohs surgery. For small superficial Mohs defects, alternatives to traditional mucosal advancement such as V-Y mucosal advancements can also be used.^9,10,13^

Our article looks at the application of the lateral myomucosal lip island flaps for more challenging small to medium mucosal and muscle defects. In comparison with existing techniques, these flaps avoid additional adjacent tissue excision. The technique preserves lower lip volume and minimizes effacement of the vermilion line. In this study, we analyzed the last 20 cases performed by the senior author where the myomucosal lip island flap was employed.

## Methods

### Study design

**Fig. 1.**
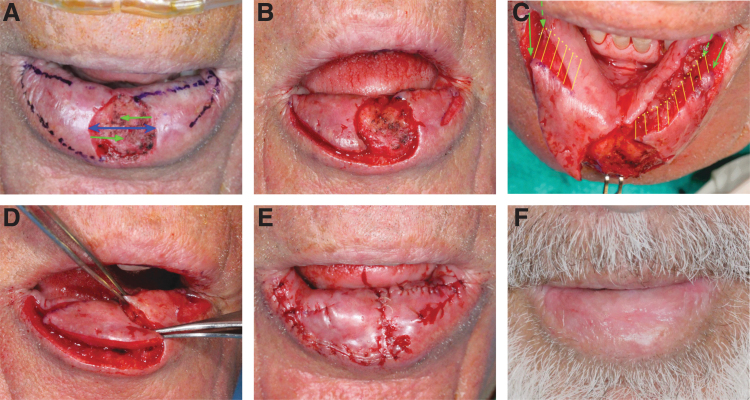
Lower lip reconstruction with myomucosal lip island flap for central defect. **(A)** Surgical planning, defect size of 1.6 × 1.1 cm, bridging distance 1.6 cm (blue arrow), advancing distances 0.8 cm (green arrows). **(B)** Incisions. **(C)** Depth of muscle release depicted by yellow lines. Solid green arrows show the superficial flap corner advancement, whereas broken green arrows show the deep flap corner advancement. **(D)** Flap advancements of 0.8 cm each. **(E)** Immediate postoperatively. **(F)** Final aesthetic outcome 7 weeks postoperatively.

IRB approval was obtained from St. Joseph Health Center for Clinical Research. A retrospective chart review was done on all patients who have had the lower lip reconstruction from 2005 to 2019 logged in a database. Waiver of informed consent was granted by the IRB committee. The study analyzes the myomucosal lip island flaps from 2014 when the senior author began employing this technique. We gathered data on the location and dimensions of the defect, the bridging distance of the defect, the advancing distance of the flaps, additional techniques employed, and indications for surgery. The overall bridging distance was defined to be the width of the defect that was closed by single or double flaps. The advancing distance was defined to be the distance each flap actually advanced (Fig. 1A). All patients were reviewed for functional outcomes, whereas 18 out of 20 were reviewed for aesthetic outcomes by the authors. Functional outcome evaluation was based on postoperative clinical notes. Aesthetic outcome assessment was based on the available postoperative photographs. Written informed consent was obtained from participants whose photographs are presented. Postoperative photographs were evaluated by the authors to determine how many cases resulted in scar visibility, vermilion retraction, contour loss, and flattening or loss of vermilion roll of the lip to determine the degree of visible deformities. Defect dimensions were recorded, but the data analysis was performed on the defect bridging distance. Only laterally advancing myomucosal flaps were included.

### Surgical technique

**Fig. 2.**
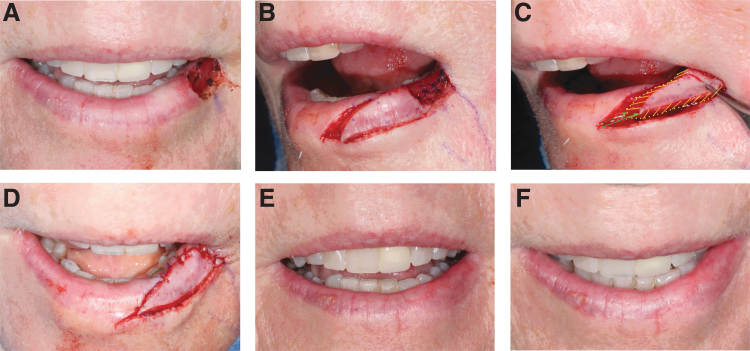
Lower lip reconstruction with myomucosal lip island flap for lateral defect. **(A)** Defect size of 1.5 × 0.9 cm, bridging distance of 1.5 cm. **(B)** Incisions. **(C)** Depth of muscle release depicted by yellow lines. Solid green arrows show the superficial flap corner advancement, whereas broken green arrows show the deep flap corner advancement. The flap advanced 1.5 cm. **(D)** Immediate postoperatively. **(E)** Anterior view of final aesthetic outcome 6 months postoperatively. **(F)** Superior view of final aesthetic outcome 6 months postoperatively.

The design of the reconstruction is based on lateral myomucosal island flap advancement (Fig. 1). The essence of this technique is volume rearrangement of the lower lip musculature. Mucosa simply follows the deep submucosal and muscle releases and movement. An initial incision is carried laterally from the anterior edge of the defect along or preferably just inside the vermilion border to avoid flattening the natural lip roll and lip fullness of scar contracture. The incision is curved away from the vermilion border toward the wet mucosa of the lip. A return incision is made from the lateral extent of the incision toward the posterior edge of the defect. The incisions are extended deep through the muscle with spreading technique, often identifying the inferior labial artery (Fig. 1C). Muscle release extends through the entire thickness of the orbicularis oris and extends into the depressor labii inferioris. The muscle in the most lateral extent of the flap is separated, releasing the flap's movement toward the defect. Thick muscle release is required and is performed in a progressive manner around the flap to minimize wound closure tension. The defect is then prepared by excising the beveled edges of a Mohs excision if needed. The leading edge of the flap is undermined for at least 5 mm from the edge, deep within the muscle. This helps eversion and fullness at the closure line. The incisions are closed in the deep muscle layer and in the superficial mucosa. The lateral lip donor site is closed in V-Y manner. Defects in the lateral third of the lower lip can be reconstructed with a single myomucosal island flap (Fig. 2).

A subtotal myomucosal island flap is identical to the island flap with the exception of a small myomucosal bridge left intact near the defect. The remnant connecting bridge is narrow, measuring 10–20% of the flap total length. The remainder of the muscle release is the same as the traditional lateral myomucosal island flap—extensive and deep, necessary to achieve volume rearrangement of the muscle.

When a subtotal island flap is used, another flap is usually required from the contralateral side of the defect. It could be another myomucosal subtotal island flap or a smaller myomucosal advancement. In some cases, a wedge of mucosa posterior to the defect is excised as a standing cone without employing a second flap. Defects in the middle third of the lower lip are reconstructed with bilateral subtotal island flaps to minimize lip shape distortion by distributing the tension and muscle volume throughout the lip (Fig. 1). The second myomucosal island flap is created by extending the incision from the posterior edge of the defect along the dry–wet lip border. The incision is carried contralaterally as far as the first flap, then returned to the anterior edge of the defect, leaving a small mucosal and muscle bridge intact.

For complex defects involving the vermilion and cutaneous portions of the lip, the red lip is reconstructed as described. The cutaneous defect is reconstructed with a separate skin flap or a graft (Fig. 4C).

## Results

**Fig. 3.**
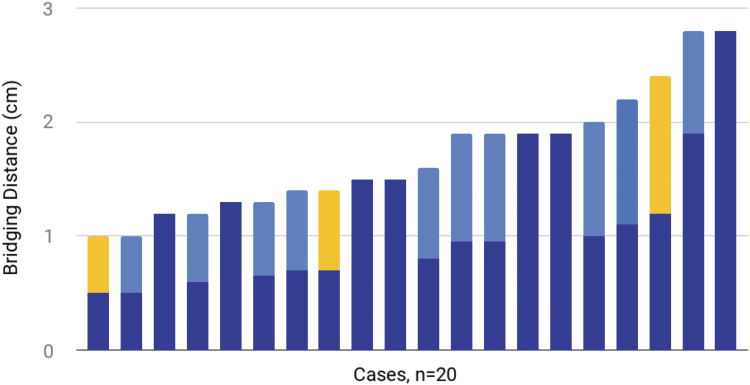
Histogram of bridging distance and individual advancing distances in the 20 cases of the myomucosal lip island flaps. Cases who employed combined techniques for red lip reconstruction are represented with two colors in the stacked bar graph. Shades of blue represent myomucosal lip island flaps and yellow represent other flaps. Cases with dark blue and light blue indicate that two myomucosal lip island flaps were employed.

Over the past 5 years, 31 myomucosal lip island flaps have been employed in 20 patients, aged 34–85 years with a mean of 62 years of age. Of the 20 patients, there was a 2:1 male to female ratio. Eighteen patients presented with squamous cell carcinoma, one with a basal cell carcinoma, and one with a hemangioma. The analysis of flap's limitations was based on the defect bridging distance and the flap advancing distance (Fig. 3). The defects were repaired with lateral flap(s) advancing over the width of the defect. The individual advancing distances describe how far each myomucosal flap actually moved. In the case of a single flap, the advancing distance equals the bridging distance. In the case of combined techniques, the two advancing distances combined equal the bridging distance. In all but one case of combined techniques, both flaps advanced over half the defect width. In one case of bilateral myomucosal lip island flaps, one advanced two-third of the width, whereas the other advanced one-third. Our definition of combined techniques only included those that were used in the red lip reconstruction and did not include the flaps used for the cutaneous defect reconstruction. The minimum and maximum bridging distances were 1.0 and 2.8 cm, respectively. The average bridging distance was 1.7 cm (standard deviation [SD] 0.5 cm). The minimum and maximum advancing distances were 0.5 and 2.8 cm, respectively. The average advancing distance was 1.0 cm (SD 0.5 cm).

Table 1 categorizes the flaps by bridging distance—the width of the defect. It breaks up the techniques based on whether a single flap was used to close the red lip defect or whether a combined two-flap technique was used. Flaps for cutaneous lip closure are not included. Out of 20 patients, 13 were treated with combined techniques as compared with 7 with a single flap, bridging distances up to 2.8 cm. Combined technique includes two myomucosal lip island flaps or a single myomucosal flap and another rotation flap or advancement flap.

**Table 1.** **Single flap and combined technique for red lip closure categorized by bridging distance**

No. of flaps used for red lip closure	Bridging distance (cm)				Total
	1.0–1.5	1.5–2.0	2.0–2.5	2.5–3.0	
Single flap	2	4	0	1	7
Combined technique	6	3	3	1	13
Total	8	7	3	2	20

The total number of cases who had one or more myomucosal lip island flaps employed is 20. Single flap indicates that only one myomucosal lip island flap was utilized. Combined techniques indicate that either two myomucosals or one myomucosal and one other flap were used.

**Fig. 4.**
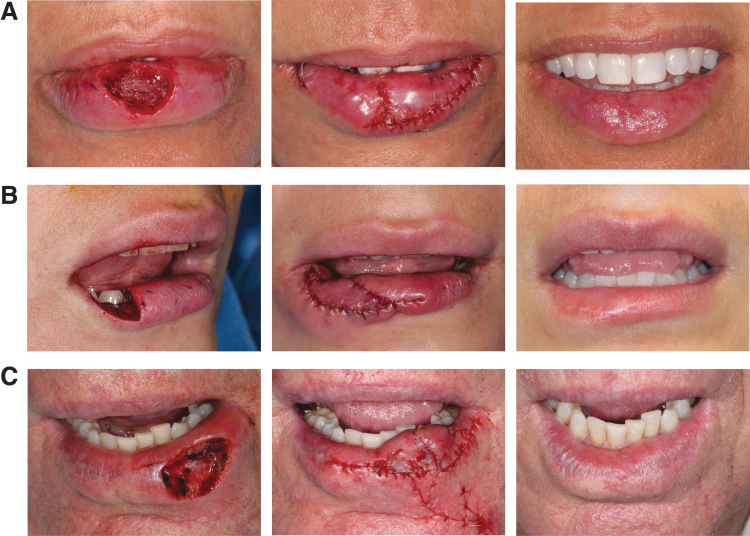
Before and after photographs. **(A)** 1.9 × 1.3 cm defect, 1.9 cm bridging distance and 0.95 cm advancing distances of each myomucosal island flap, and final aesthetic outcome 3 weeks postoperatively. **(B)** 2.8 × 1.6 cm defect, 2.8 cm bridging distance and 2.8 cm advancing distance of a single myomucosal island flap, and final aesthetic outcome 6 months postoperatively. **(C)** 2.4 × 1.3 cm defect, 2.4 bridging distance and 1.2 cm advancing distances (a separate cutaneous partial island flap for the cutaneous part of the defect), and final aesthetic outcome 3 months postoperatively.

There were no functional complications or permanent sensory deficits in the 20 cases. Complications included one patient (5%) who developed a mucocele that required excision and one patient (5%) who developed dehiscence in the lateral lip. Available postoperative photographs permitted aesthetic evaluation of 18 of the 20 cases (Fig. 4). Aesthetic complications included four cases (22%) of mild vermilion line retraction, one case (6%) of mild contour irregularity, and one case (6%) of visible white scar on red mucosa.

## Discussion

Lower lip reconstruction algorithms routinely mention a series of progressive techniques with increasing size of the defect. For small to medium defects, those techniques have been limited to mucosal advancement or wedge excision with primary closure. Deep lip muscle defect repair can also include vertical V-Y myocutaneous advancements from the cutaneous lip and chin.^11,12^ However, those techniques are mostly applied to larger defects. Alternatives to mucosal advancement flap have included primary closure without undermining for vermilionectomy defects.^14^ In that article, Barry described a simpler alternative to wide submucosal undermining achieving similar results. Smaller superficial vermilion defects can also be addressed with linear closure or secondary intention healing, both risking lip contour distortion.^9,10,15^ V-Y mucosal advancements both laterally^9^ and vertically^13^ can also be used for smaller superficial defects. Although they are tissue sparing, these flaps risk lip distortion due to localized tension effect. This, in part, accounts for the popularity of wide mucosal advancement—achieving a uniform lip contour.

It is important to differentiate the mucosal V-Y advancement and hatchet flap from the myomucosal lip island flaps. Although similar on the surface, the former separates the tissue superficially, whereas the myomucosal island flap requires a deep and extensive muscle release (Figs. 1C and 2C). Such release must be based on specific vascular supply to avoid the risk of flap ischemia. The flap muscle is then reattached to new anchor points. The clear advantage of myomucosal lip island flap with such release is wide distribution of tension and wide redistribution of volume defect.

The results of the traditional techniques have been adequate and sometimes excellent, especially in the elderly patients with thin pliable lips. However, they can leave an aesthetic impact that is visible and suboptimal, especially in the younger patients. In our study, half of the patients (10) were between 34 and 60 years old. This age group requires a technique with lip contour preservation.

Horizontal lip shortening and vermilion line effacement are the primary negative consequences of traditional reconstructive techniques of the lower lip. Raschke quantified that effect with cephalometric analysis.^7^ Vermilion line lip roll effacement is least tolerated in the central lip, whereas the lateral lip is often flat naturally and is not as affected by lip shortening techniques.

Tissue economy is the hallmark of island flaps. The lateral myomucosal island flap avoids additional muscle and mucosal tissue loss, achieving a high level of lip aesthetic. Island flap by its design distributes the defect across a much larger area, avoiding a localized impact of a tissue defect.

In 15 of the 20 patients, we used bilateral subtotal island flaps to redistribute the defect over a larger volume of two flaps than a single island flap would provide. Five of our patients were reconstructed with a total island flap. For larger central defects, we prefer bilateral subtotal island flaps. For lateral defects, total island flaps are optimal. The small myomucosal bridge of the subtotal island flap does little to limit the deep flap release. Limiting the surface release of the subtotal flap is possible because of mucosal elasticity. This affords surface incision efficiency when used with another flap. Total island flaps can be used in all the cases where subtotal island flaps were used. In cases of inelastic mucosa, full mucosal incisions were required, creating a total island flap. The goal is to minimize surface incisions and utilize pliability of the mucosa. So although extensive muscle release is required for volume redistribution, surface incisions can be minimized.

Of our 20 patients, combined techniques were used on both sides of 13 defects. Bilateral subtotal island flaps were used in 10 of the 13 combined flaps, whereas the remaining 3 used a myomucosal island flap with a mucosal advancement or rotation flaps. Bilateral myomucosal subtotal island flaps were mostly used for central lower lip defects. We believe that the most critical central lower lip defects do best with the bilateral technique. The two flaps most efficiently distribute the tension and the volume loss across the lower lip.

We found that any extension of the red lip defect into the cutaneous lip required a separate reconstruction in three patients of the combined technique group. The partial inferior cutaneous island flap was our preferred technique for reconstruction of the cutaneous portion of the lower lip defect. In those patients, where the cutaneous defect was not addressed separately (four patients), the vermilion line retracted the exact distance as the original cutaneous defect portion (a few millimeters in our patients).

Up to 2.8 cm, defects were successfully repaired with myomucosal lip island flaps. No flap vascular compromise occurred, demonstrating the robust blood supply of the flaps despite aggressive release.

The flaps' mobility was defined by the flap advancing distance. It is an important measure of a flap's limitation.^16^ We have introduced the concept of defect bridging distance to define the maximal linear dimension that can be closed with one or two flaps. Thus, defect bridging distance is a more specific subset of the defect dimensions. Flap advancing distance is always a subset of the defect bridging distance.

A limitation to the myomucosal lip island flap is the temporary sensation of numbness at the incision site. Most patients reported little to no numbness within 1 year after surgery. The lips demonstrated a partial return of sensation within a few months of repair. Complete return of sensation occurred over a period of 6–9 months in most patients. The assessment of sensation outcome is limited to patient self-reporting and not by any objective criteria.

Another limitation of the study involves the assessment of aesthetic results. The analysis was performed by the authors on the 18 cases who had both pre- and postoperative photographs.

Small to medium lower lip defects up to one-third of the lip width can be effectively treated with lateral myomucosal lip island flaps, resulting in excellent cosmetic outcomes. This technique achieves lip volume preservation and minimizes lip roll effacement, supplanting the need for mucosal advancement and wedge resection in many cases. The aggressive release of the myomucosal island flap is at the core of that outcome while reliably preserving the vascular supply.
